# Evaluation of multifaceted interventions on antibiotic prescribing in community-acquired pneumonia

**DOI:** 10.1017/ash.2026.10333

**Published:** 2026-05-25

**Authors:** Kristen South, Stephanie Ducas, Ashley Cubillos, Elisabeth Chandler, Elena Gatskevich

**Affiliations:** Pharmacy, Lee Health: Lee Memorial Health System, Fort Myers, USA

## Abstract

**Objective:**

To evaluate the impact of systemwide antimicrobial stewardship interventions on antibiotic prescribing patterns for community-acquired pneumonia(CAP) in a community health system.

**Design:**

Retrospective pre-post implementation study.

**Setting:**

Adult acute care hospitals in a community health system.

**Patients:**

Adults 18 years or older admitted to an acute care hospital with a diagnosis of CAP identified by ICD-10 codes.

**Methods:**

This retrospective, pre-post implementation study was completed at four adult acute care hospitals in a community health system. Antimicrobial stewardship interventions targeted toward CAP were rolled out between July 2020 and March 2021. Data to capture prescribing habits was collected pre and postimplementation. The inclusion criteria were patients who were at least 18 years old, admitted to an adult acute care hospital, and diagnosed as CAP based on ICD-10 code, while exclusion criteria were patients who were transferred from another hospital, had a pneumonia with an onset of 48 hours or later after admission, or had a concomitant non-respiratory tract infection during receipt of antibiotics for CAP. The primary outcome was percentage of patients who received empiric anti-MRSA or antipseudomonal antimicrobial coverage for CAP in accordance with current guideline recommendations.

**Results:**

Four hundred patients were included in the analysis, with 207 patients in the preimplementation arm and 193 patients in the postimplementation arm. The percentage of patients who received appropriate empiric coverage significantly increased from 34.7% in the preimplementation group to 60.1% in the postimplementation group (*P* < .0001).

**Conclusions:**

Multifaceted antimicrobial stewardship interventions significantly improve guideline-concordant empiric MRSA and *P. aeruginosa* antimicrobial coverage.

## Introduction

Community-acquired pneumonia (CAP) is one of the leading causes of morbidity and mortality worldwide and is associated with significant costs to the healthcare system.^1,3^ The ubiquity of CAP in the hospitalized patient population highlights the importance of strong antimicrobial stewardship in this disease state. Magill et al., found that nearly 80% of patients hospitalized for CAP in the United States had antibiotics prescribed that were unsupported by best clinical evidence.^2^ Overutilization of antibiotics, including excessive spectrum and length of treatment, leads to extended hospital stays, increased adverse effects, and antimicrobial resistance.^3,4^

The 2019 update of the IDSA and American Thoracic Society (ATS) CAP guidelines highlight several clinical scenarios with potential antimicrobial stewardship opportunities. The term “healthcare-associated pneumonia” (HCAP) was abandoned, and an emphasis was instead placed on identifying risk factors for multidrug-resistant organisms (MDROs), reserving empiric antibiotic coverage for methicillin-resistant *Staphyloccocus aureus* (MRSA) or *Pseudomonas aeruginosa* for select patients. Additionally, a strong recommendation was made for antibiotic duration for uncomplicated CAP to be limited to 5 days in the absence of clinical instability (eg, fever, elevated white blood cell count, elevated heart rate and/or respiratory rate, hypotension, hypoxemia, altered mental status, and lack of appetite) at end of treatment.^5^ In 2025, the ATS released additional guidelines further recommending a reduced course of antibiotics for clinically stable outpatients or inpatients, with a minimum of 3 days duration, rather than a full five day course. For inpatients with severe CAP, these guidelines reflect the 2019 guidelines recommendation for a 5-daycourse of treatment.^6^

Several recommendations for antimicrobial stewardship that may be applied to CAP are featured in the 2016 Infectious Disease Society of America (IDSA), Society for Healthcare Epidemiology of America (SHEA), and Centers for Disease Control (CDC) Antimicrobial Stewardship Program guidelines, including development of facility-specific treatment guidelines for common infections, such as CAP; though evidence is overall of low quality.^8,9^ Nonetheless, promising improvements seen in a limited number of retrospective studies after implementation of CAP-specific guidelines and order sets include a reduction in hospital length of stay and duration of antimicrobial therapy without adverse effects on clinical outcomes.^5,10,11^

**Figure 1.**
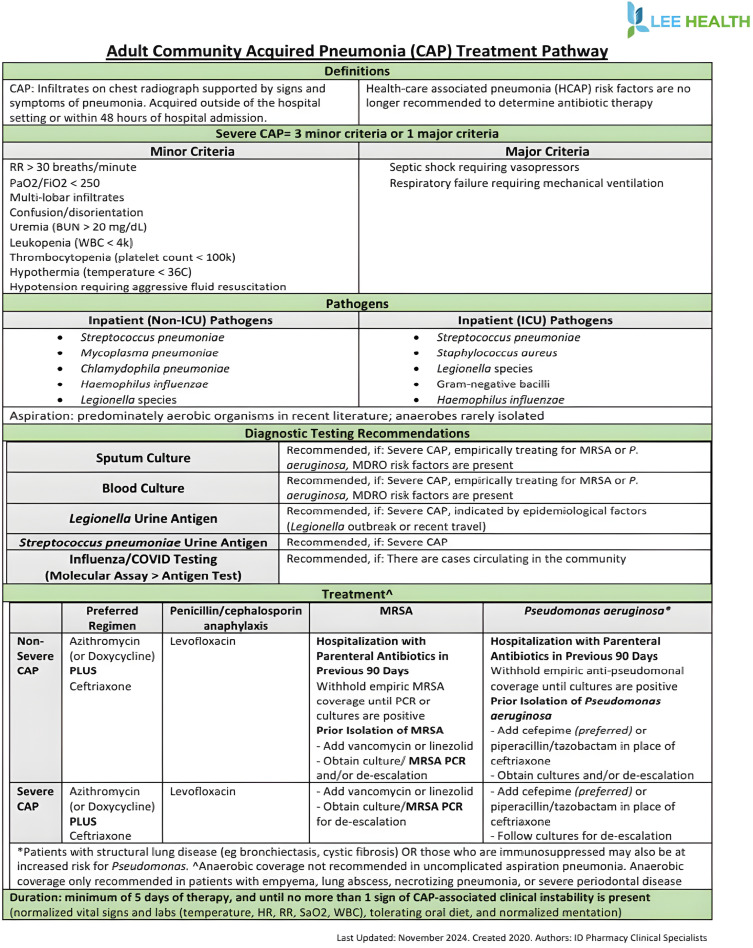
CAP treatment pathway.

Internal data from our health system indicated that a significant number of patients in our hospitals were being treated for CAP with unnecessary *P. aeruginosa* and MRSA empiric antibiotic coverage according to the 2019 IDSA/ATS CAP guidelines and receiving total durations of antibiotic therapy that exceeded guideline recommendations. Based upon this information, our system’s Antimicrobial Stewardship Committee implemented several interventions aimed at improving antibiotic prescribing for CAP, including a CAP treatment pathway document (Figure 1) and CAP order set revisions that outlined pneumonia severity criteria and guided providers on when broader coverage may be indicated Education on the updated CAP guidelines was provided through a CME session for prescribers, and CAP was designated as the 2021 annual pharmacist competency to ensure systemwide dissemination and standardization of practice (November 2020—December 2021).The objective of this retrospective review was to assess the efficacy of these antimicrobial stewardship interventions made by evaluating changes in antibiotic prescribing patterns in CAP patients, before and after implementation.

## Methods

### Setting and study population

This retrospective, quasi-experimental study was conducted across four adult acute care hospitals in a community health system. Patients were included if they met the following criteria: at least 18 years old, admitted to an adult acute care hospital and diagnosed with CAP based on ICD-10 code (Table 1). Patients were excluded if they transferred from another hospital, had a pneumonia with an onset of 48 hours or later after admission, or had a concomitant non-respiratory tract infection during receipt of antibiotics for CAP. Patients admitted from July 1^st^, 2019, to June 30^th^, 2020, constituted the preimplementation group, while patients admitted from April 1^st^, 2021, to September 30^th^, 2021, constituted the postimplementation group. This study was approved by our organization’s Institutional Review Board.

**Table 1.** Diagnosis codes

ICD-10 diagnosis code	Diagnosis
J09.X1	Influenza due to identified novel influenza A virus with pneumonia
J10.0X	Influenza due to other identified influenza virus with pneumonia
J11.0X	Influenza due to unidentified influenza virus with pneumonia
J12.XX	Viral pneumonia, not elsewhere classified
J13	Pneumonia due to *Streptococcus pneumoniae*
J14	Pneumonia due to *Hemophilus influenzae*
J15.XXX	Bacterial pneumonia, not elsewhere classified
J16.X	Pneumonia due to other infectious organisms, not elsewhere classified
J17	Pneumonia in diseases classified elsewhere
J18.X	Pneumonia, unspecified organism

### Outcome measures

The primary outcome was the percentage of patients empirically receiving anti-MRSA or antipseudomonal antimicrobials concordant with current guideline recommendations. Guideline-concordant indications for empiric use of anti-MRSA or antipseudomonal antimicrobials in CAP were defined as prior respiratory culture isolation of MRSA or *P. aeruginosa* at any point; or meeting severe CAP criteria plus having a history of hospitalization within the previous 90 days with parenteral administration of antimicrobials. Anti-MRSA antibacterial agents are defined as the following: ceftaroline, vancomycin, telavancin, dalbavancin, oritavancin, clindamycin, linezolid, tedizolid, minocycline, tigecycline, or trimethoprim-sulfamethoxazole (TMP-SMX). Antipseudomonal agents are defined as the following: piperacillin-tazobactam, ceftazidime, cefepime, meropenem, aztreonam, ciprofloxacin, levofloxacin, tobramycin, gentamicin, amikacin, ceftazidime-avibactam, or ceftolozane-tazobactam. Per the IDSA criteria, severe CAP was defined as requiring either mechanical ventilation or use of vasopressor, or meeting 3 minor CAP criteria.

Secondary outcomes include duration of antimicrobial therapy, percentage of patients with opportunity for de-escalation, duration of IV antibiotic therapy, percentage of patients receiving at least 24 hours of anti-MRSA antibacterial agents, and percentage of patients receiving at least 24 hours of antipseudomonal antibacterial agents, both including and excluding levofloxacin as an antipseudomonal agent in the analysis. De-escalation was defined as a reduction of the number of anti-MRSA or antipseudomonal antimicrobials in a patient’s regimen between the empiric and definitive regimens. An opportunity for de-escalation was defined as one of the following during admission: identification of non-MRSA or non-*Pseudomonas* culture (blood or respiratory) that would allow de-escalation of empiric antimicrobials, or a negative MRSA nasal swab collected within 48 hours of starting anti-MRSA antimicrobial therapy.

### Statistical analysis

Statistical analyses were performed using JMP (14). Categorical data was analyzed using the χ^2^ Test and presented using percentages. Continuous, non-parametric data were analyzed using the Mann-Whitney U Test and presented as median (25–75% interquartile range). Continuous, parametric data were analyzed using the Student t-Test. Additionally, a descriptive analysis for the postimplementation arm was performed to determine the overall percentage of patients who received empiric antibiotic orders from the newly-implemented CAP order sets (emergency department and/or inpatient admission), and the percentage of empiric antibiotic coverage chosen from the CAP order sets in concordance with the 2019 IDSA/ATS CAP guideline risk factors for MRSA and *P. aeruginosa*. An estimated 390 patients needed to be included in the chart review to achieve a power of 80 percent to detect a 10 percent difference in the primary outcome of percentage of patients who received empiric anti-MRSA or antipseudomonal antimicrobials for CAP in accordance with current guideline recommendations.

## Results

**Figure 2.**
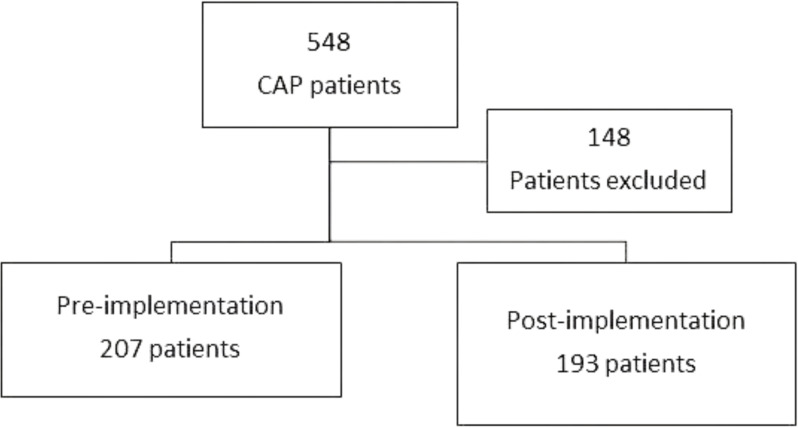
Patient screening diagram.

A total of 548 patients were reviewed during the study period, with 148 of these patients excluded. Common reasons for exclusion include onset of pneumonia greater than 48 hours from admission, concomitant non-respiratory tract infections, and transfers from outside facilities (Figure 2).

Four hundred patients were included in the analysis, with 207 patients in the preimplementation arm and 193 patients in the postimplementation arm. Table 2 describes the demographics of the patient population. There were no statistically significant differences in age or gender, with an average age of 72 years in the preimplementation arm compared to 71 years in the postimplementation arm (*P* = .38), and both groups being 48% male (*P* = .99). While there were slight differences in CAP severity minor criteria, the overall incidence of severe CAP was similar between groups, at less than 10%. No patients in either arm required vasopressor use or mechanical ventilation.

**Table 2.** Baseline demographics

	Pre (n = 207)^a^	Post (n = 193)^a^	P-value
Age (years); mean ± SD^b^	72.00 + 14.41	70.69 + 15.07	.38
Gender (male)	101 (48.7)	94 (48.7)	.99
CAP^c^ severity criteria (within 24 hours of admission)			
RR^d^ > 30 breaths/minute	19 (9.2)	1 (0.5)	<.001
PaO2/FiO2 ratio^e^ < 250	6 (2.9)	44 (23)	<.001
BUN^f^ > 20 mg^g^/dL^h^	94 (46)	72 (37)	.08
WBC^i^ < 4 x 10^9^/L	12 (5.8)	5 (2.6)	.15
Platelet < 100 x 10^9^/L^j^	9 (4.3)	2 (1)	.06
Temperature < 36°C^k^	3 (1.4)	1 (0.5)	.62
Multi-lobar infiltrates	87 (42)	109 (57)	<.05
Documented confusion	6 (3)	11 (5.7)	.17
Fluid bolus for SBP^l^ < 90 mmHg^m^	11 (5.3)	0	<.001
Vasopressor use	0	0	–
Mechanical ventilation	0	0	–
Severe CAP^n^	18 (8.7)	12 (6.3)	.23
MDRO^o^ risk factors			
Prior documented respiratory MRSA^p^	8 (3.8)	1 (0.5)	.04
Prior documented respiratory	2 (1.0)	1 (0.5)	.33
MRSA in previous 365 days			
Prior documented respiratory *P. aeruginosa*	6 (2.9)	2 (1.0)	.29
Prior documented respiratory *P. aeruginosa* in previous 365 days	2 (1.0)	1 (0.5)	.43
Documented hospitalization w/ IV^q^ antibiotics in previous 90 days	32 (15.5)	34 (17.6)	.56
Respiratory culture collected	83 (40.1)	63 (32.6)	.04
MRSA growth	1 (0.5)	3 (1.6)	.31
*P*. *aeruginosa* growth	7 (3.4)	5 (2.6)	1.00
MRSA PCR^r^ collected	54 (26.1)	54 (28.0)	.62
Positive result	3 (1.4)	5 (2.6)	.72
Respiratory viral panel collected	159 (76.8)	130 (67.4)	.34
SARS-CoV-2 identified	5 (2.4)	3 (1.6)	.51
Viral Etiology identified	35 (16.9)	21 (10.9)	.09
Specialty consults during admission			
Infectious disease	57 (27.5)	34 (17.6)	.02
Pulmonology	103 (49.8)	71 (36.8)	.01

a All values expressed as n (%) unless otherwise noted; ^b^SD, standard deviation, ^c^CAP, community acquired pneumonia, ^d^RR, respiratory rate, ^e^PaO2/FiO2, partial pressure of oxygen/fraction of inspired oxygen, ^f^BUN, blood urea nitrogen, ^g^mg, milligram, ^h^dL, deciliter, ^i^WBC, white blood count, ^j^L, liter, ^k^°C, degrees Celsius, ^l^SBP, systolic blood pressure, ^m^mmHg, millimeters of mercury, ^n^Severe CAP = vasopressor use, mechanical ventilation, OR 3 minor criteria, ^o^MDRO, multi-drug resistant organisms, ^p^MRSA, methicillin-resistant *Staphylococcus aureus*, ^q^IV, intravenously, ^r^PCR, polymerase chain reaction.

Both arms had a low incidence overall of prior documented respiratory MRSA (3.8% pre vs .5% post) and *P. aeruginosa* (2.9% pre vs 1.0% post). Growth of either organism from respiratory cultures during the index admission was infrequent and similar between groups. Positive MRSA nasal swabs were also infrequent in both arms (1.4% pre vs 2.6% post.) A minority of our patients presented with SARS-CoV-2 (2.4% pre vs 1.6% post).

The primary outcome, receipt of guideline-concordant empiric MRSA and *P. aeruginosa* antimicrobial coverage, significantly improved from 34.7% in the preimplementation group to 60.1% in the postimplementation group (*P* < .001). When examining utilization of broad-spectrum agents as empiric therapy, the percentage of patients who received at least 24 hours of anti-MRSA agents decreased from 31.4% to 22.7% between preimplementation and postimplementation, respectively (*P* = .05). The percentage of patients receiving at least 24 hours of antipseudomonal agents, also decreased from 60.4% in preimplementation to 44.5% in postimplementation (*P* < .05). When excluding levofloxacin as an antipseudomonal agent, there was no statistically significant difference in antipseudomonal agent use between groups (Table 3).

**Table 3.** Primary and secondary outcomes

	Pre (n = 207)^a^	Post (n = 193)^a^	P-value
Guideline-concordant empiric ^b^MRSA and *P. aeruginosa* antimicrobial coverage	72 (34.7)	116 (60.1)	<.001
Received ≥ 24 hours of anti-MRSA antibacterial agents during admission	65 (31.4)	44 (22.7)	.05
Received ≥ 24 hours of antipseudomonal antibacterial agents during admission	125 (60.4)	86 (44.5)	<.05
Received ≥ 24 hours of antipseudomonal antibacterial agents during admission, *excluding* levofloxacin	85 (41.1)	70 (36.3)	.37
Duration of antimicrobial therapy (days); mean ± SD^c^	8.71 ± 3.45	8.22 ± 2.88	.12
Duration of IV^d^ antibiotic therapy (days); median [IQR]^e^	4 [3–5]	4 [3–5]	.04
Duration of outpatient antimicrobial therapy, (days); mean ± SD	5.28 ± 2.41	5.18 ± 2.24	.72
Opportunity for therapeutic de-escalation based on respiratory or blood cultures	45 (21.7)	28 (14.5)	.06
Opportunity for therapeutic de-escalation based on MRSA PCR^f^	27 (13.0)	21 (10.9)	.51
De-escalation performed independently by prescriber	55 (26.6)	45 (23.3)	.45
De-escalation performed as recommended by pharmacist	8 (3.9)	6 (3.1)	.68

a All values expressed as n (%) unless otherwise noted, ^b^MRSA, methicillin-resistant *Staphylococcus aureus*, ^c^SD, standard deviation, ^d^IV, intravenous, ^e^IQR, interquartile range [25% – 75%], ^f^PCR, polymerase chain reaction.

There were limited opportunities for therapeutic de-escalation in both groups based on microbiology results, with less than 22% of patients in either group having opportunities for de-escalation based on results of respiratory culture or blood culture, and only up to 13% having an opportunity based on a negative MRSA nasal swab result. Both groups had similar rates of MRSA nasal swab collection (26.1% vs 28%). There was no change in overall duration of therapy between groups, with a mean total duration of therapy exceeding 8 days. When evaluating duration based on phase of care, there was no difference in duration between groups for inpatient duration of therapy or outpatient duration of therapy. Both the preimplementation and postimplementation groups had an average inpatient duration of therapy of greater than 4 days, while the duration of therapy prescribed at discharge exceeded 5 days for both groups.

## Discussion

In this multicenter, prepost implementation study, antimicrobial stewardship interventions targeted toward CAP were associated with a significant increase in guideline-concordant empiric antibiotic therapy. This suggests that a multifaceted approach to CAP antimicrobial stewardship has the potential to improve antibiotic prescribing for CAP within community health systems.

Previous studies evaluating CAP-targeted antimicrobial stewardship have focused predominantly on duration of therapy, through implementation of order sets and/or clinical practice guidelines at their institutions.^12,13^ With our interventions, the emphasis was placed on validating appropriate empiric coverage to reduce broad-spectrum use, which was a unique aspect compared to previous literature on CAP-targeted stewardship. Haas et al created CAP-specific clinical practice guidelines and an order set for their institution, which recommended guideline-directed therapy of ceftriaxone and azithromycin for course of 5 days for non-severe CAP patients, unless a severe penicillin allergy existed, in which levofloxacin was recommended. With their interventions, they only evaluated duration of therapy and did not focus on reduction in broad-spectrum agents beyond levofloxacin.^10^ While drug allergy-related prescribing choices were not specifically assessed in our study between pre and postgroups, potential reasons for changes in levofloxacin ordering might include de-emphasis of this agent in treatment pathway/order sets and clearer allergy-related guidance in the order set. Similarly, Gordon et al found that duration of therapy was reduced after implementation of an institution-specific CAP clinical practice guideline. In their guideline, they also recommended ceftriaxone and azithromycin unless a severe penicillin allergy existed, for a duration of 5 days in the setting of non-severe CAP, with an evaluation of patients at the 48-hour mark for a transition to oral therapy if improving, or the need to broaden therapy if no improvement.^10^ Similar to our study, both of these trials evaluated duration of therapy for inpatient, outpatient and total. Despite having similar interventions to these studies, we did not see any improvement in any phase of our duration of therapy. Given that our inpatient duration of therapy was generally appropriate, there is likely opportunity for enhanced optimization of antibiotic prescribing at transitions of care within our health system.

Our total duration of therapy exceeded the guideline recommendations by over 3 days in both the preimplementation and postimplementation groups, with patients receiving over 8 days. Overprescribing of antibiotics is not a novel concept, with previous studies supporting excessive total duration of therapies ranging from 8 to 10 days.^12,13^ A major area for opportunity identified with this study revolves around transitions of care, as patients were discharged with a full course (>5d) of antimicrobial therapy for CAP after receiving nearly a full course inpatient ( > 4 d). Limited studies evaluating antimicrobial stewardship at time of discharge have been completed. Yogo et at implemented a prospective audit at time of discharge with recommendations to providers for duration of therapy for common infections, including CAP, and found a significant reduction in duration prescribed at discharge, but no difference in total duration of therapy.^13^ Additionally, Caplinger et al evaluated the effect of implementing a pharmacist-led protocol targeting duration of antimicrobial therapy for CAP patients and found a reduction in excessive durations of therapy prescribed at discharge without an impact on readmissions.^14^ Our institution utilizes pharmacists for medication reconciliation at time of discharge, but future efforts on recommendations for length of antimicrobial therapy could be valuable.

While our order sets and clinical practice guidelines did recommend the shortest effective treatment durations, the main emphasis was evaluating patients for MDRO risk factors and determining the need for broad-spectrum agents as empiric therapy. The IDSA/ATS CAP guidelines recommend coverage for MRSA and/or *P. aeruginosa* in the setting of previous respiratory isolation of these organisms, severe CAP with recent hospitalization with parenteral IV antibiotic administration, or if there are locally validated risk factors present. The previous categorization of healthcare-associated pneumonia (HCAP) has been abandoned in attempt to reduce the overutilization of anti-MRSA and antipseudomonal agents. With locally validated risk factors, clinicians should evaluate their local incidence of MDRO pathogens and overall resistance patterns. At our institution, we have an antibiogram that is updated annually to guide clinicians in appropriate antimicrobial agents based on resistance patterns.

This study had several strengths. The interventions targeting CAP were practical, with relatively low time-intensity requirements to implement for the realized impact on the health system. While these interventions served as a supplement to a more preferred method of antimicrobial stewardship, prospective audit and feedback, which has stronger, higher quality evidence, but is more labor intensive on a daily basis, they could serve as an alternative or supplemental measure for smaller hospitals with limited resources.^7^ Additionally, this study had a small percentage of patients with severe CAP, previous history of MDROs, and positive MDRO cultures. This supports generalizability of streamlining interventions to a non-severe CAP population in many community hospitals and health systems across the country with an overall low incidence of MDROs.

This study was limited by the fact that it was a retrospective study and, therefore, the data is limited to the accuracy of the electronic health record. While data on antibiotic prescribing could be accurately collected, patient adherence after discharge could not, which may impact the true total duration of therapy. Additionally, given that the primary aim of the study was to evaluate guideline-concordant prescribing, clinical outcomes, such as mortality, readmissions, or complications, were not evaluated. Data collection for the preintervention did include the initial phase of the COVID-19 pandemic. Intangible prescriber-specific effects on empiric antimicrobial prescribing during this time frame for patients presenting with pneumonia symptoms cannot be completely excluded, though these factors may also have been present in the postgroup given inclusion of the Delta COVID-19 period. Although peak pneumonia season typically occurs during winter and was included in the preintervention period, the postperiod also represented a high CAP burden, and given comparable rates of CAP severity, COVID-19 positivity, and viral etiologies, seasonal variation was unlikely to have significantly influenced treatment patterns. Lastly, because this initiative evaluated antimicrobial selection according to CAP guideline recommendations rather than clinical outcomes, data on immunocompromised status and chronic lung disease were not collected, as the study population primarily represented a community health system with predominantly immunocompetent patients. Therefore, the findings may be less generalizable to tertiary or specialty centers with a higher proportion of immunocompromised or otherwise complex patient populations.

Overall, this study demonstrated a positive impact of multifaceted stewardship interventions on improving guideline-adherence to empiric therapy for CAP at a community hospital, and reduction of broad-spectrum antimicrobial use. Future directions for antimicrobial research at a local level should evaluate specific strategies to improve duration of therapy.
